# Comparative Evaluation of Glucose Levels in Gingival Crevicular Blood and Peripheral Blood Using Two Different Glucometers in Patients Suffering From Periodontal Disease

**DOI:** 10.7759/cureus.104414

**Published:** 2026-02-27

**Authors:** Barsha Nath, Alpana Talukdar, Maitreyee Sharma, Rakesh K Medhi, Putul Mahanta

**Affiliations:** 1 Department of Dentistry, Nalbari Medical College and Hospital, Nalbari, IND; 2 Department of Paediatrics, Nalbari Medical College and Hospital, Nalbari, IND; 3 Department of Periodontics, Regional Dental College, Guwahati, IND; 4 Department of Forensic Medicine and Toxicology, Nalbari Medical College and Hospital, Nalbari, IND

**Keywords:** capillary blood, diabetes mellitus, glucometer, periodontal disease, random blood sugar

## Abstract

Introduction: Given dentists' increasing role in healthcare, dental clinics may be essential sites for diabetes screening. Gingival bleeding is a hallmark of periodontal inflammation. Gingival crevicular blood (GCB) collection with a portable glucometer for glucose measurement is a safer, more convenient, patient-friendly, and time-saving option. The primary objective of the current investigation was to compare random blood glucose levels with those in gingival crevicular and peripheral blood, as determined using two different glucometers. The study's secondary goal was to assess the feasibility and inter-glucometer agreement of estimating blood sugar from gingival crevicular blood using a glucometer as a chairside diabetes screening tool.

Methods: The present study included 85 patients with bleeding during probing in the maxillary anterior region. The GCB sample was collected from an appropriate site of the maxillary anterior teeth. Peripheral blood (PB) samples were collected from the patients' right index fingers to determine PB glucose levels. Blood glucose levels were measured using glucometers from two different brands: Dr. Morepen (A) and Contour Plus (B). Venous blood samples were used to determine random blood sugar (RBS) level using laboratory methods. The data were divided into five groups: GCB A: GCB evaluated by glucometer A; GCB B: GCB evaluated by glucometer B; PB A: PB evaluated by glucometer A; PB B: PB evaluated by glucometer B; and RBS: random blood sugar.

Bland-Altman analysis was used for intergroup comparisons. Concordance correlation analysis across groups was performed to assess the concordance between two variables. Intraclass correlation was used to assess absolute agreement among the different methods of blood glucose measurement. A p-value of less than 0.05 was considered significant.

Results: The present study comprised 85 participants. The participants' ages ranged from 15 to 70 years. The majority, 53 (62.4%) of the participants, were male. Almost 94% (n=80) of the participants were normoglycemic. The mean glucose level was highest in the PB group (102.68 mg/dL) and lowest in the GCB B group (99.58 mg/dL). There was no significant systematic bias between GCB A with RBS (mean difference = -0.23, 95% CI: -2.22 to 1.77) and PB B (mean difference = 0.09, 95% CI: -1.84 to 2.02), whereas PB A exhibited systematic overestimation. Furthermore, RBS (mean difference=0.88, 95% CI: -0.91 to 2.66), PB B (mean difference=1.20, 95% CI: -0.60 to 3.00), and GCB A (mean difference=1.11, 95% CI: -0.42 to 2.63) are less biased than GCB B. Both GCB A and GCB B showed significant concordance with RBS, PB A, and PB B (correlation coefficients >0.95, p-values <0.001).

The two glucometers under study showed almost perfect absolute agreement in estimating blood sugar levels from gingival crevicular blood (ICC single-measure value > 0.9) and peripheral blood (single-measure value > 0.9).

Conclusion: GCB oozing from periodontal pockets during routine examination may be used for glucose estimation as precisely as that from peripheral and venous blood. There is no statistically significant difference in glucose estimation results obtained with the two glucometers used in the study. GCB can serve as a source for noninvasive glucose monitoring in a dental setting.

## Introduction

Diabetes mellitus (DM) is a complex metabolic disease characterized by elevated blood sugar levels resulting from a malfunction in insulin action, secretion, or both [1]. It is associated with a wide range of micro- and macrovascular complications, altered wound healing, and periodontitis [2,3]. People with type 2 diabetes, formerly known as adult-onset diabetes or non-insulin-dependent diabetes, have insulin resistance and typically have a relative insulin deficit [4]. Periodontitis is among the most frequent consequences of DM. Severe periodontitis is about three times as common in those with poorly managed diabetes as in people without the disease [5]. Patients with periodontitis should be screened for DM and vice versa because there is a positive bidirectional link between the two conditions [6]. Periodontal disease is one of the two most significant oral illnesses contributing to the worldwide burden of chronic disease, making it a serious global public health problem [7].

Over the past three decades, India's diabetes burden has significantly increased. With an annual percentage change of 0.63%, the incidence of diabetes rose from 162.74 to 264.53 cases per 100,000 between 1990 and 2021 [8]. Research has revealed that Indians possess several unusual characteristics, including low risk thresholds for diabetes susceptibility. Compared to other populations, Indians acquire diabetes at a younger age and with lower body weights. Indians are genetically predisposed to diabetes, and the aforementioned causes negatively impact them [9]. Early detection of diabetes is a primary health care priority. Diabetes is typically diagnosed by measuring capillary blood glucose levels with a reflectance-based blood glucose meter, followed by confirmatory diagnostic testing. Traditional laboratory methods for detecting blood glucose are time-consuming and require sophisticated equipment. The introduction of blood glucose monitors has enabled clinicians to test blood glucose at the chairside. In contrast to the laboratory procedure, results are obtained rapidly, allowing the doctor to make immediate judgments [10].

Given the growing importance of dentists in healthcare, dental offices could be crucial sites for detecting diabetes. Gingival bleeding, a hallmark of periodontal inflammation, has a favorable profile for use as a blood glucose test [11]. In individuals with moderate to severe periodontitis, routine pocket probing during a periodontal examination yields adequate GCB. The collection of GCB using a portable glucometer for glucose estimation is a more practical, safe, patient-friendly, and time-saving alternative and might therefore help increase chair-side diabetes screening [12]. Even in the case of very low gingival crevicular bleeding, glucose can be measured with the self-monitored device. In general, the accuracy of these novel glucometers has been reliable. They can also be used to monitor blood glucose levels in patients with known diabetes [13].

Furthermore, there is a greater likelihood of a favorable prognosis of periodontitis when diabetes is discovered in the early stages [14]. It is known that during diagnostic procedures, periodontal inflammation, with or without DM, results in a large amount of extravasated blood. During a periodontal examination, routine probing is less stressful and more familiar to the practitioner than puncturing the finger with a sharp lancet. Glucose meters make it painless to check for blood leaking from patients' gingival crevices during routine periodontal exams. They may also be a straightforward and reasonably priced in-office screening tool for any patient suspected of having diabetes.

In the present study, an effort was made to measure blood glucose levels in GCB using a non-invasive technique. Venous and PB were collected as references for comparison with the GCB in estimating blood glucose levels. The primary objective of the present study was to compare blood glucose levels in gingival crevicular blood and peripheral blood, measured with two different glucometers, with random blood sugar levels. The secondary objective of the study was to evaluate inter-glucometer agreement and the feasibility of estimating blood glucose from gingival crevicular blood using a glucometer for chairside screening of diabetes.

## Materials and methods

The present comparative study was conducted at the Department of Periodontology and Implantology, Buddha Institute of Dental Sciences and Hospital (BIDSH), Patna, among patients attending the Department of Periodontology and Implantology OPD at BIDSH who had bleeding on probing in the maxillary anterior region. The subjects were chosen regardless of sex, religion, or socioeconomic status. The patient's general and detailed medical, family, and personal history were recorded, and the clinical examination was performed. All the collected data was entered into the specially designed proforma. Periodontal conditions were assessed for every tooth present. Periodontal probing depth (PPD) and clinical attachment level (CAL) were measured with William's graduated probe and recorded. To avoid inter-examiner variation, all subjects were examined by a single operator. Following the clinical examinations, all patients were prepared to collect blood samples to estimate their blood glucose levels. Written informed consent was obtained from each participant after the procedure was explained. The Institutional Ethical Committee of Buddha Institute of Dental Sciences & Hospital, Patna, Bihar, approved the study.

Inclusion criteria

Any patient aged 15 years or above with or without diabetes reporting to the OPD of the Department of Periodontology and Implantology, BIDSH, in whom there was bleeding on probing from the maxillary anterior region, and those giving voluntary consent to participate in the study were included.

Exclusion criteria

Patients with a history of prolonged usage of any anticoagulants were excluded.

Sample size

The sample size was calculated using the formula for determining the required sample size to test whether a correlation coefficient differs from zero. The minimum sample size required for the study was n=85, based on an expected correlation coefficient of r=0.30 between the two techniques, with a 95% confidence interval and 80% power.

Sample collection

The GCB sample was collected from an appropriate site of the maxillary anterior teeth. For each patient, only the bleeding site during probing was selected. Sites with suppuration were excluded from the study. The selected site was isolated with gauze or cotton rolls. William's graded probe was used to check the interdental papilla between the incisors or between the incisors and canines, and the gingival crevice was checked for bleeding after removal. Blood glucose levels were measured using glucometers from two different brands: (A) Dr. Morepen (Morepen Laboratories Ltd., Gurgaon, India) and (B) Contour Plus (Bayer, India). After placing the strips into the glucometer, the test ends were brought into contact with the bleeding site to obtain a blood sample on the test strip without disturbing the gingival or palatal tissues.

The test strips were held until the device beeped, and the meter counted down for 8 seconds. Later, the blood glucose level was recorded in mg/dL. Following glucose measurement in GCB, capillary blood samples were collected from the patients' right index fingers to determine PB glucose. The soft tissue surface of the finger was cleaned with a 10% isopropanol solution. The finger was then punctured with a sterile disposable lancet, allowing a drop of blood to form. The first drop of blood was discarded, and as soon as the second drop appeared, the test end of the strip was brought into contact with the bleeding site and held until the instrument beeped, indicating that the meter had counted down to 8 seconds. The blood glucose measurement was then recorded in mg/dl. Venous blood samples were collected from each patient's antecubital vein via venipuncture using a 2 ml syringe to determine their RBS level. Patients were instructed to squeeze their fists until the vein was perceptible. A tourniquet was placed around one to two inches above the antecubital fossa. Blood was withdrawn after cleaning the puncture site with a 10% isopropanol solution. To prevent blood from leaking, the patients were instructed to apply gentle finger pressure to the site for a few minutes. After collection, samples were sent to the library of BIDSH, Patna.

Statistical analysis

MS Office Excel Sheet version 2019 (Microsoft Redmond Campus, Redmond, Washington, United States) and IBM Corp. Released 2016. IBM SPSS Statistics for Windows, Version 22. Armonk, NY: IBM Corp. were used for data compilation and analysis. For categorical data, descriptive statistics such as frequencies and percentages were used to describe the distribution. Mean, standard deviation, and standard error were used to represent continuous distributions. The Shapiro-Wilk test was used to confirm the normality of the numerical data. Bland-Altman analysis was used for intergroup comparisons. Concordance correlation analysis across groups was performed to assess the concordance between two variables. Intraclass correlation was used to assess absolute agreement among the different methods of blood glucose measurement. A p-value of less than 0.05 was deemed statistically significant.

## Results

The present study comprised 85 participants. The participants' ages ranged from 15 to 70 years, with a mean (±standard deviation) of 35.49 (±13.98 years), and most (n = 37, 43.5%) were in the 15-30 age group. The majority (53, 62.4%) of patients were male. The random blood sugar levels ranged from 69.7 mg/dL to 396.7 mg/dL. Almost 94% of the participants were normoglycemic (n=80). Generalized chronic gingivitis was the most common clinical diagnosis among 43 (50.6%) participants (Table 1).

**Table 1:** Profile of participants (N=85) The data were represented as frequency and percentage.

Variables	Categories	Frequency (n=85)	Percentage
Age	15-30 years	37	43.5
	31-45 years	25	29.4
	>45 years	23	27.1
Gender	Female	32	37.6
	Male	53	62.4
Diabetes status	Normoglycemic	80	94.1
	Pre-diabetic	4	4.7
	Diabetic	1	1.2
Clinical diagnosis	Generalized chronic gingivitis	43	50.6
	Generalized chronic periodontitis	20	23.5
	Generalized chronic gingivitis with localized chronic periodontitis	19	22.4
	Generalized chronic gingivitis with inflammatory enlargement	3	3.5

Blood glucose levels in GCB and PB were evaluated using Dr. MorepenR (A) and Contour PlusR (B) glucometers. Also, the RBS was evaluated with venous blood using laboratory methods. For statistical analysis, the data were divided into five groups: GCB A: GCB evaluated by glucometer A; GCB B: GCB evaluated by glucometer B; PB A: PB evaluated by glucometer A; PB B: PB evaluated by glucometer B; and RBS: random blood sugar.

The mean glucose level was highest in the PB A group (102.68 mg/dL) and lowest in the GCB B group (99.58 mg/dL). The mean glucose levels estimated by glucometer B were lower in both GCB and PB than those estimated by glucometer A (Table 2).

**Table 2:** Mean blood glucose levels among different groups (N=85) The data were presented as mean ± standard deviation (Std. Dev.). GCB A: Gingival crevicular blood evaluated by glucometer A, GCB B: Gingival crevicular blood evaluated by glucometer B, PB A: Peripheral blood evaluated by glucometer A, PB B: Peripheral blood evaluated by glucometer B, RBS: Random blood sugar

Group	N	Mean ± Std. Dev.	Std. Error	95% Confidence
				Lower Bound-Upper Bound
GCB A	85	100.68±36.48	3. 96	92.81-108.55
GCB B	85	99.58±36.96	4.01	91. 60-107.55
PB A	85	102.98±35.01	3. 80	95.42-110.53
PB B	85	100.78±35.09	3. 81	93.21-108.34
RBS	85	100.45±37.14	4.03	92.44-108.46

**Figure 1:**
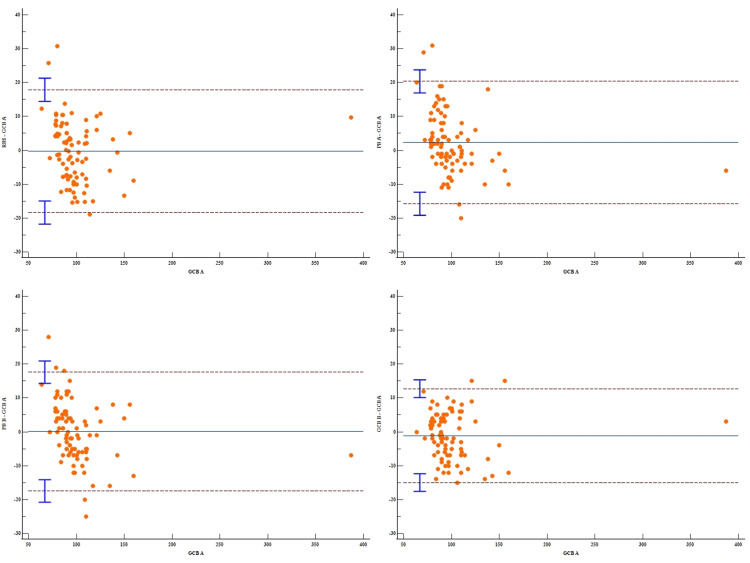
Bland–Altman plots comparing GCB A with the other methods GCB A: Gingival crevicular blood evaluated by glucometer A, GCB B: Gingival crevicular blood evaluated by glucometer B, PB A: Peripheral blood evaluated by glucometer A, PB B: Peripheral blood evaluated by glucometer B, RBS: Random blood sugar

As seen from Table 3, no significant systematic bias was observed between GCB A with PB B (mean difference = 0.09, 95% CI: -1.84 to 2.02) and RBS (mean difference = -0.23, 95% CI: -2.22 to 1.77). Compared to GCB A, PB A showed a systematic overestimation of measurements. In the case of GCB B, the mean blood glucose level was slightly lower than that of GCB A. However, the difference was statistically not significant. Thus, GCB A showed good agreement with RBS, PB B, and GCB B (Figure 1).

**Table 3:** Comparison of GCB A with other methods (Bland–Altman analysis) GCB A: Gingival crevicular blood evaluated by glucometer A, GCB B: Gingival crevicular blood evaluated by glucometer B, PB A: Peripheral blood evaluated by glucometer A, PB B: Peripheral blood evaluated by glucometer B, RBS: Random blood sugar

Reference	Method	n	Diff (Method-Reference)			Limits of Agreement			
			mean	SD	95% CI	Lower	95% CI	Upper	95% CI
GCB A	RBS	85	-0.23	9.25	-2.22 to 1.77	-18.35	-21.78 to -14.93	17.90	14.47 to 21.32
	PB A	85	2.29	9.21	0.31 to 4.28	-15.77	-19.18 to -12.36	20.36	16.95 to 23.77
	PB B	85	0.09	8.95	-1.84 to 2.02	-17.44	-20.75 to -14.13	17.63	14.32 to 20.94
	GCB B	85	-1.11	7.07	-2.63 to 0.42	-14.96	-17.58 to -12.35	12.75	10.14 to 15.37

**Figure 2:**
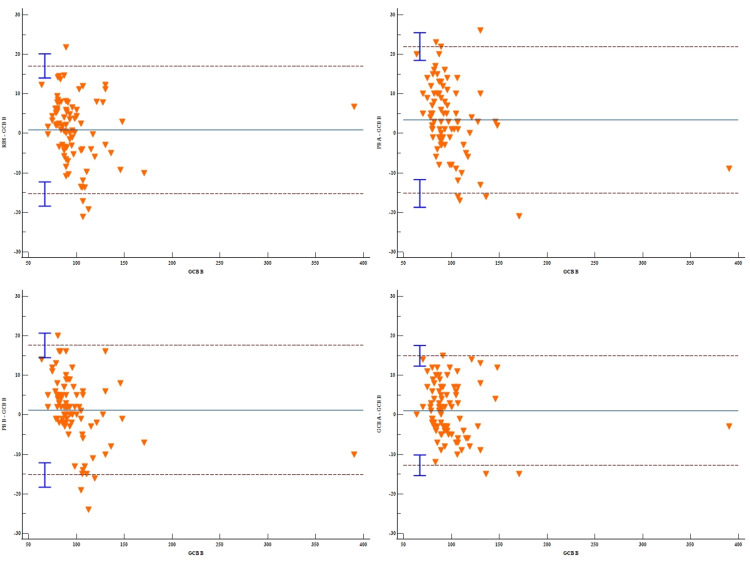
Bland–Altman plots comparing GCB B with the other methods GCB A: Gingival crevicular blood evaluated by glucometer A, GCB B: Gingival crevicular blood evaluated by glucometer B, PB A: Peripheral blood evaluated by glucometer A, PB B: Peripheral blood evaluated by glucometer B, RBS: Random blood sugar

As seen from Table 4, RB S (mean difference=0.88, 95% CI: -0.91 to 2.66), PB B (mean difference=1.20, 95% CI: -0.60 to 3.00), and GCB A (mean difference=1.11, 95% CI: -0.42 to 2.63) exhibit minimal bias compared to GCB B. On the other hand, PB A showed a higher positive bias (≈3.4 mg/dL) than GCB B, which was statistically significant. Thus, GCB B showed good agreement with RBS, PB B, and GCB A (Figure 2).

**Table 4:** Comparison of GCB B with other methods (Bland–Altman analysis) GCB A: Gingival crevicular blood evaluated by glucometer A, GCB B: Gingival crevicular blood evaluated by glucometer B, PB A: Peripheral blood evaluated by glucometer A, PB B: Peripheral blood evaluated by glucometer B, RBS: Random blood sugar

Reference	Method	n	Diff (Method-Reference)			Limits of Agreement			
			mean	SD	95% CI	Lower	95% CI	Upper	95% CI
GCB B	RBS	85	0.88	8.27	-0.91 to 2.66	-15.34	-18.40 to -12.28	17.09	14.03 to 20.16
	PB A	85	3.40	9.48	1.35 to 5.44	-15.19	-18.70 to -11.68	21.99	18.48 to 25.50
	PB B	85	1.20	8.36	-0.60 to 3.00	-15.19	-18.29 to -12.10	17.59	14.50 to 20.69
	GCB A	85	1.11	7.07	-0.42 to 2.63	-12.75	-15.37 to -10.17	14.96	12.35 to 17.58

Both GCB A and GCB B showed substantial concordance with RBS, PB A, and PB B (concordance correlation coefficient > 0.95, p-value < 0.001). GCB A and GCB B showed the highest concordance (Table 5).

**Table 5:** Concordance correlation analysis between different groups GCB A: Gingival crevicular blood evaluated by glucometer A, GCB B: Gingival crevicular blood evaluated by glucometer B, PB A: Peripheral blood evaluated by glucometer A, PB B: Peripheral blood evaluated by glucometer B, RBS: Random blood sugar; *** Highly significant correlation with p-value < 0.001

Group		RBS	PB A	PB B	GCB B
	Sample size	85	85	85	85
GCB A	Concordance correlation coefficient	0.968	0.965	0.969	0.981
	p-value	<0.001***	<0.001***	<0.001***	<0.001***
	95% CI	0.952 - 0.979	0.947 - 0.977	0.953 - 0.979	0.971-0.988
	Pearson ρ	0.969	0.968	0.969	0.981
	Bias correction factor	0.999	0.997	0.999	0.999
GCB B	Concordance correlation coefficient	0.975	0.961	0.972	
	p-value	<0.001***	<0.001***	<0.001***	
	95% CI	0.961 - 0.983	0.941-0.974	0.958 - 0.982	
	Pearson ρ	0.975	0.967	0.974	
	Bias correction factor	0.999	0.994	0.998	

As shown in Table 6, the intraclass correlation analysis indicated that the two glucometers under study demonstrated almost absolute agreement in estimating blood sugar levels from gingival crevicular blood (ICC single-measure value > 0.9) and peripheral blood (single-measure value > 0.9). Also, all five methods showed absolute agreement, with an average inter-class correlation of 0.994 (95% CI: 0.992 to 0.996).

**Table 6:** Intraclass correlations, two-way mixed analysis for absolute agreement A p-value < 0.05 was considered significant. GCB A: Gingival crevicular blood evaluated by glucometer A, GCB B: Gingival crevicular blood evaluated by glucometer B, PB A: Peripheral blood evaluated by glucometer A, PB B: Peripheral blood evaluated by glucometer B, RBS: Random blood sugar

Variable		Intraclass correlation	95% Confidence Interval
GCB A vs. GCB B	Single measures	0.981	0.971 to 0.988
	Average measures	0.990	0.985 to 0.994
PB A vs. PB B	Single measures	0.973	0.957 to 0.983
	Average measures	0.986	0.978 to 0.991
GCB A GCB B PB A PB B RBS	Single measures	0.972	0.961 to 0.980
	Average measures	0.994	0.992 to 0.996

## Discussion

DM is a prevalent condition that impacts people globally, including in India. Low- and middle-income countries (LMICs) have the highest rates of undiagnosed DM, with over half of all people with diabetes being ignorant of their illness [15]. Impaired wound healing associated with DM poses a substantial medical, societal, and economic burden [16]. This is especially concerning in dental clinics, where most patients undergo intraoral surgical procedures regularly. Often, people visiting a dental clinic are unaware of their diabetic status, and the dental surgeon is the first to detect it. There is compelling evidence that DM and glycemic control levels affect the prevalence and severity of periodontitis [17].

These days, many dentists outfit their clinics with glucometers so they can determine a patient's blood glucose level right before surgery. Dental fear or anxiety affects a large portion of the population, which results in avoidance and/or difficulty in accepting dental care [18]. Thus, for certain anxious patients in the dental clinic, even the simple act of pricking a finger to measure the capillary glucose level using a glucometer can become a traumatic experience. Gentle probing causes bleeding in patients with periodontal disorders. The glucometer can be used to measure blood sugar levels instantly by using the blood that emerges from the gingival fissure after probing in a patient with periodontal disease. Extravasation of blood during a periodontal examination is painless, atraumatic, and unnoticed by the patient. Earlier studies have documented that GCB obtained during a diagnostic periodontal examination can be an important source for glucometer analysis and an easy-to-use, safe, and comfortable method for the patient [19]. The present study aimed to assess the correlation between GCB glucose levels and finger capillary blood glucose levels measured with glucometers and to establish the reliability of glucometer readings by comparing them with laboratory results.

The current study has not relied on results from any specific glucometer, as readings may vary across different models. A random survey was conducted in prominent local pharmacies and among patients in and around Patna to determine which glucometers to use in the present study. It was found that Dr. Morepen and Contour Plus glucometers were the most frequently used glucometers in the study area. These glucometers were both electrochemical and met the requirements of the International Organization for Standardization (ISO) 15197:2013. For the current investigation, these two glucometers have been utilized for blood sugar estimation. No statistically significant difference in mean glucose levels was observed between these two glucometers in the gingival crevicular fluid or PB.

Previous studies have documented no substantial difference in glucose estimation from GCB and PB [20,21]. In the present study, the blood sugar levels in GCB, as measured by both glucometers, showed good agreement with those in intravenous blood, as determined by the laboratory method. Similar findings were observed in various other studies [20-23]. However, systematic overestimation was observed with PB A compared with GCB A and GCB B. Although the mean blood glucose level estimated by GCB B was somewhat lower than that of GCB A, both showed high concordance (concordance correlation coefficient = 0.981, p-value < 0.001). According to the intraclass correlation analysis, the two glucometers under investigation could estimate blood sugar levels from gingival crevicular blood (ICC single-measure value >0.9) and peripheral blood (ICC single-measure value >0.9) with nearly perfect agreement. Overall, the groups showed nearly perfect agreement (interclass correlation single measure > 0.8; p < 0.001).

Even though gingival blood was first suggested as a chairside approach to diabetic screening in 1969, it was not widely adopted because previous glucometer systems required 5-6 microliters of blood. Nonetheless, most newly created glucometers require only a very small volume of blood, as little as 1 microliter, and typically produce results in 5 seconds. Thus, in recent years, there has been a resurgence of interest in this topic. The glucometers utilized in this study are from later generations and measure a tiny volume of blood in just 5 seconds. According to a recent review, glucometers used for professional purposes met ISO 15197 accuracy requirements but not clinical validity standards, whereas devices used for home use did not meet either requirement. Compared with home-use glucometers, professional-use glucometers showed higher accuracy [24].

The two glucometers used in the present study provided statistically reliable estimates of blood glucose levels from gingival crevicular blood. However, blood sugar estimation from gingival crevicular blood should be done with caution, as saliva, crevicular fluid, plaque, and inflammatory exudate can be isolated with cotton rolls alone, which is suboptimal due to potential contamination. The diagnostic potential of these biofluids is affected by how they are collected [25]. Dilutional effects and inflammatory exudate can have a major impact on blood glucose levels [26], as in both cases, the glucose readings might be lower. Dilutional effects might make it difficult to determine whether low glucose is due to an infection or to fluid excess.

Most research in this area has been done on individuals with DM. Without considering the patients' diabetes status, Singh S et al. examined the effectiveness of GCB, finger capillary blood, and plasma glucose level in measuring blood glucose levels in patients with chronic periodontitis. The current study was also carried out on a similar cohort of patients, and both investigations have produced comparable results [27]. The present study included all patients with bleeding on probing in the maxillary anterior region, irrespective of their diabetes status. The clinical utility of screening tools depends on performance across glycemic ranges. In the present study, 94.1% (n=80) of the study participants were normoglycemic, which might artificially inflate the correlation and agreement. However, the mean glucose levels estimated by both glucometers for the only known diabetic participant in the present study were within acceptable limits, as seen from the Bland-Altman plots.

Limitation

Serum glucose, still considered the gold standard for estimating blood glucose levels, is measured by conventional laboratory methods. In contrast, glucometers measure whole blood glucose. Therefore, if we perform both tests simultaneously, the self-monitoring devices are likely to show a lower value than the laboratory results [28]. However, the present study did not take this into account, which may be considered a limitation. Blood collection from the gingival crevice during probing may be contaminated by saliva, plaque, and dental debris. The current investigation took every possible precaution to prevent such contamination. Although unlikely, the operating area's isolation with only cotton rolls in place meant such risks remained possible. This is yet another study drawback. If we adequately address the limitations, we can plan future research with a larger sample.

## Conclusions

Glucose estimation done from GCB is as effective as that done from PB and venous blood. Compared with venous blood, there was no substantial bias in glucose values obtained from gingival crevicular blood and peripheral blood using glucometers. However, using glucometers to estimate glucose levels from peripheral blood resulted in a slight overestimation. When assessing blood sugar levels from gingival crevicular blood, the two glucometers under investigation demonstrated nearly perfect agreement. Thus, GCB can effectively be used for noninvasive glucose monitoring in a dental care setting.
